# Effects of Underwater Friction Stir Welding Heat Generation on Residual Stress of AA6068-T6 Aluminum Alloy

**DOI:** 10.3390/ma15062223

**Published:** 2022-03-17

**Authors:** Hassanein I. Khalaf, Raheem Al-Sabur, Mahmoud E. Abdullah, Andrzej Kubit, Hamed Aghajani Derazkola

**Affiliations:** 1 Mechanical Department, Engineering College, University of Basrah, Basrah 6100, Iraq; hassanein.khalaf@uobasrah.edu.iq (H.I.K.); raheem.musawel@uobasrah.edu.iq (R.A.-S.); 2 Mechanical Department, Faculty of Technology and Education, Beni-Suef University, Beni-Suef 62511, Egypt; iec.mahmoud@gmail.com; 3 Department of Manufacturing and Production Engineering, Faculty of Mechanical Engineering and Aeronautics, Rzeszow University of Technology, Al. Powst. Warszawy 8, 35-959 Rzeszów, Poland; akubit@prz.edu.pl; 4 Department of Mechanics, Design and Industrial Management, University of Deusto, Avda Universidades 24, 48007 Bilbao, Spain

**Keywords:** thermo-mechanical simulation, underwater friction stir welding, residual stress measurement, friction stir welding

## Abstract

This article aims to study water-cooling effects on residual stress friction stir welding (FSW) of AA6068-T6 aluminum alloy. For this reason, the FSW and submerged FSW processes are simulated by computational fluid dynamic (CFD) method to study heat generation. The increment hole drilling technique was used to measure the residual stress of welded samples. The simulation results show that materials softening during the FSW process are more than submerged. This phenomenon caused the residual stress of the joint line in the submerged case to be lower than in the regular FSW joint. On the other hand, the results revealed that the maximum residual stresses in both cases are below the yielding strength of the AA6068-T6 aluminum alloy. The results indicated that the residual stress along the longitudinal direction of the joint line is much larger than the transverse direction in both samples.

## 1. Introduction

After introducing the friction stir welding (FSW) process, various lightweight structures have been produced that could not be produced before [1,2]. The welding heat in this process is generated by friction at the contact surface between workpieces and tools [3]. Thus, this process is placed in solid-state joining techniques. The rotational FSW tool produces frictional heat and soft surrounding materials [4]. The FSW tool traverses along the weld line and stirs the softened materials for mixing and joining. FSW as a solid-state process has many benefits over conventional and high-power welding techniques. With this welding process, mostly non-weldable alloys will be weldable [5]. This technique provides a convenient solution for welding such alloys and improves joining processes in various environments [6]. Many alloys can be joined in air, various temperature water, and vacuum environments [7]. Underwater friction stir welding (UFSW) is newly developed for offshore and marine structures [8]. The UFSW process has the following main stages: plunge, dwell, traverse, and exit [9,10]. In the first stage, the process tool is slowly plunged into the sheets to be welded until it reaches the required depth. It is held in position for a short time while still rotating, called the dwelling stage [11,12]. In this stage, the material is held stationary before the tool starts to be preheated before the tool starts moving forward [13]. During the next step, the tool traverses along the joint line. In this stage, the materials ahead of the tool are extruded into the stir zone (SZ) and fill the joint line. When the welding process is finished, the UFSW tool exits from the joint line. This step is the final stage of the joining process [14].

Until now, a couple of researchers reported the effects of residual stress on the FSW joint. Castro et al. [15] studied the residual stress of T-joints of FSW AA5065 aluminum alloy and AA7075 aluminum alloy. They showed that the residual stress of the heat-affected zone (HAZ) and thermo-mechanical affected zone (TMAZ) could be modeled by a logarithmic model. They showed that the residual stress concentrates on the flange side in T-joint structures. Jamshidi et al. [16] simulated FSW of AA6061 and AA5086 aluminum alloys. They measured residual stress of the produced joint by center-hole drilling (CHD) method. They found a relation between the FSW process parameters and residual stress of the joint line. They showed that the FSW tool rotational velocity affects the maximum residual stress, and FSW tool traverse velocity affects residual stress distribution. Jamshidi [17] also simulated the FSW process of AA6082-T6 and AA7070-T6 aluminum alloys and measured the residual stress of the joint by X-ray. 

Ting [18] used the CHD method to measure the residual stress in a 3 mm AA2024 aluminum sheet welded by the FSW process. He showed that the residual stress in the longitudinal direction was more than transverse residual stress. Ripley and Linton [19] applied neutron diffraction to measure residual stress of 7XXX aluminum alloy and indicated that the residual stress in the joint line decreased over time. There has been no report regarding the effects of submerged FSW on residual stress of the joint line until now. For this reason, this article investigates the effects of submerging with considering heat generation on residual stress of AA6068 aluminum alloy.

## 2. Materials and Methods

The AA6068-T6 aluminum sheets as raw materials were cut into 4 mm × 150 mm × 100 mm pieces. The sheets’ mechanical properties are shown in Table 1. The welding procedure was done with a modified milling machine (T-34, Xu-Kay, Xulian, China).

**Figure 1:**
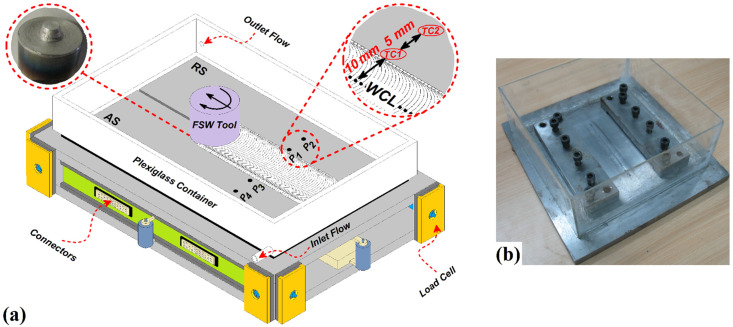
(**a**) Schematic view of welding setup, thermocouples placement, and FSW tool. (**b**) Underwater friction stir welding setup.

A stainless steel-made flexible fixture was used for fixing the aluminum sheets in a suitable place during the FSW process. The FSW procedure is done with the submerging condition as underwater friction stir welding (UFSW) and conventional FSW process. Plexiglas surrounds the welding setup to be filled with water and installs onto an FSW machine (Bayer, Berlin, Germany). There was one inlet and outlet valve to allow water to flow during the welding procedure. This issue aimed to maintain a constant heat transfer coefficient between the weld line and water. The diameter of the inlet and outlet valves was constant, and the volumetric flow rate (Debi) of water was 5 mm^3^/s. For the UFSW case, the workpieces and tools were submerged during the welding process. The water temperature was 30 °C during tests. After several experimental tests, the water temperature being between 20 and 40 °C, the best cooling rate was obtained at 30 °C. This paper aims to not affect various cooling rates on the properties of the joint line. The welding was performed using a tungsten carbide FSW tool with a frustum probe. The tool shoulder had 18 mm diameter. The tool pin had 4 mm upper diameter with a 30-degree draft and 3.2 mm length. The picture of the used tool in this study is depicted in Figure 1a. During the joining process, welding tool angular velocity, forward velocity, tilt angle, and plunge depth were 920 rpm, 45 mm/min, 2°, and 0.1 mm, respectively. Four K-type thermocouples (Omega, Norwalk, CT, USA) were inserted at different locations, as shown in Figure 1a. Two thermocouples were placed on the advancing side (AS) and two on the retreating side (RS). 

Two places were selected in AS and RS. The places are named P1 and P2 in the AS, and P3 and P4 in the RS. P1 and P2 had 10 mm and 15 mm distances from the weld centerline (WCL). Furthermore, P1 and P2 had 10 mm and 15 mm distances from the WCL. The thermocouples that were placed in P1, P2, P3, and P4, were respectively named TC1, TC2, TC3, and TC4. The results of thermocouples output are reported with mentioned name in the Results and Discussions Section. The maximum temperature was recorded and presented for residual stress evaluation. The used UFSW setup is depicted in Figure 1b.

**Figure 2:**
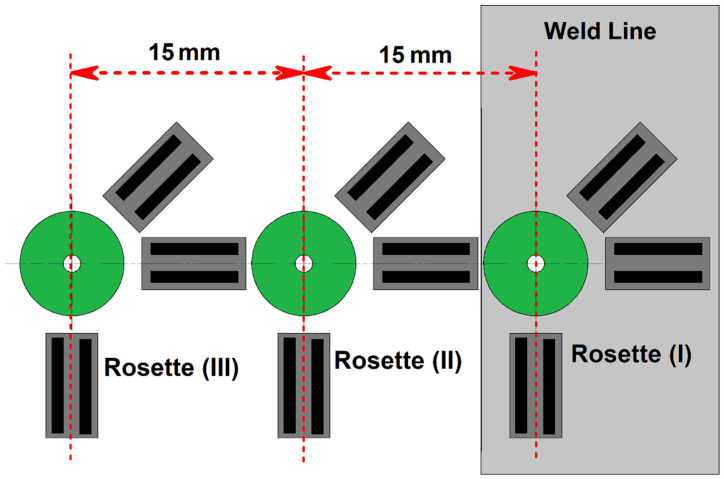
Schematic view of rosette placement on joint line.

The center-hole drilling (CHD) technique is employed for measuring residual stress. At the early stage (after the FSW process), three surface strain gauge rosettes (FRS-2-23, TML, Sint Technology, Calenzano, Italy) were placed on the joint line surface. The strain gauge rosette was glued to the FSW joint line and the lead wires were attached. The strain gauge rosette had three measuring grids (0°, 45°, 90°) with a nominal resistance of 120 Ω. The measuring grids had a circular pattern according to the ASTM E837 standard. Next, the CHD machine (SINT MTS 3000, Sint Technology, Calenzano, Italy) was aligned with the surface strain gauge rosette. The CHD machine is equipped with an air compressor to increase the drilling speed to 300,000 rpm. Then, the center of the rosette is drilled with 0.8 mm drill hole diameter and 3.2 mm drill hole depth. The depth and strain gauge data were then analyzed to calculate residual stress distribution. Schematic view of rosette placement on the joint line depicted in Figure 2. The collected strains from strain gauges are put into the equations below [20,21]:

(1) $$p=\frac{\varepsilon_{1}+\varepsilon_{3}}{2}$$

(2) $$q=\frac{\varepsilon_{3}-\varepsilon_{1}}{2}$$

(3) $$t=\frac{\varepsilon_{1}+\varepsilon_{3}-2\varepsilon_{2}}{2}$$

In Equations (1)–(3) the *ε* are the released strains from the rosette. In the next step we need to calculate the stresses [20,21]:

(4) $$P=\frac{\sigma_{1}+\sigma_{2}}{2}$$

(5) $$Q=\frac{\sigma_{2}-\sigma_{1}}{2}$$

(6) $$T=\tau_{13}$$

Then, the relation between stresses and strains with calibration coefficients can be calculated as [20,21]:

(7) $$\bar{a}P=\frac{Ep}{1+\nu }$$

(8) $$\bar{b}Q=Eq$$

(9) $$\bar{b}T=Et$$

*E* and *υ* are Young modulus and Poisson coefficient, respectively. *a* and *b* are calibration coefficients and *σ* and *τ* represent normal and shear stresses. With reached stresses, the stress elasticity equations for each layer (*k* index) can be calculated as [21,22]:

(10) $${\left(\sigma_{\max}\right)}_{k,}{\left(\sigma_{\min}\right)}_{k}=P_{k}\pm \sqrt{Q_{k}{}^{2}+T_{k}{}^{2}}=E\left(\frac{P}{\bar{a}\left(1+\nu \right)}\pm \frac{\sqrt{q^{2}+t^{2}}}{\bar{b}}\right)$$

(11) $$\beta k=\frac{1}{2}\tan^{-1}\left(\frac{T_{k}}{Q_{k}}\right)=\frac{1}{2}\tan^{-1}\left(\frac{t}{q}\right)$$

## 3. Process Modelling

### 3.1. Model Description

A 3D steady-state coupled material flow and heat model was used in the present research. The commercial ANSYS FLUENT software under the computational fluid dynamics (CFD) package was used to complete the simulation process. According to the actual experimental tests, the dimension of workpieces and geometry FSW tools were selected [23]. In the simulation domain, the origin point is set at the middle of the tool, the x-axis represents the welding direction, and the normal axis of the welding tool is set to the z-axis. The welding tool had an angular velocity, and the FSW tool traveling speed controlled the interior domain setting. Both bottom and top workpiece’s side walls have the same velocity as the inlet. To avoid the reverse flow, the outlet of the simulation domain was set at zero pressure [24]. Single-phase flow conservation equations for momentum, energy, and continuity were used to simulate this process. To model the quasi-static thermal and fluid flow boundary problem outside the interface, a non-Newtonian single-phase fluid was assumed for the workpieces [25]. The mass conservation and energy conservation are presented as Equations (12) and (13), respectively:

(12) $$\frac{du}{dx}+\frac{dv}{dy}+\frac{dw}{dz}=0$$

(13) $$\rho c\left(\frac{\partial T}{\partial t}+u\frac{\partial T}{\partial x}+v\frac{\partial T}{\partial y}+w\frac{\partial T}{\partial z}\right)=k\left(\frac{\partial^{2}T}{\partial x^{2}}+\frac{\partial^{2}T}{\partial y^{2}}+\frac{\partial^{2}T}{\partial z^{2}}\right)$$

Due to heat generation of the tool being higher than vaporization of water, local water vaporization around the tool may happen. For this reason, the appropriate water flow and boundary condition were selected [26].

### 3.2. Weld Metal Model

The strain rate tensors and deviatoric stress were correlated using the aluminum alloy AA6068-T6 as a non-Newtonian fluid. Thermo-mechanical properties based on temperature and density were used. Non-Newtonian viscosity of aluminum alloy was assumed as temperature and strain rate dependent. For this reason, the viscosity of aluminum alloy as a function of strain rate and flow stress is presented by [27,28]:

(14) $$\psi =\frac{F_{s}}{3\dot{\varepsilon }}$$

In Equation (14), *F_s_* represent flow stress of aluminum alloy and can be presented as [29]:

(15) $$F_{s}=\frac{1}{\alpha }\sinh^{-1}{\left(\frac{Z}{A}\right)}^{\frac{1}{n}}=\frac{1}{\alpha }\left({\left(\frac{Z}{A}\right)}^{\frac{1}{n}}+\left(1+{\left(\frac{Z}{A}\right)}^{\frac{2}{n}}\right)\right)$$

In Equation (15), the *Z* represents the Zener–Holloman parameter used for the calculation of the temperature-dependent strain rate [30]:

(16) $$Z=\dot{\varepsilon }\exp\left(\frac{Q}{RT}\right)$$

The curve fitting of the hot compression test of AA6068-T6 aluminum alloy is used to obtain the material constitutive constants. In this study, the results from the hot compression test of AA6068-T6 aluminum alloy for curve fitting, were obtained from published results from Memon et al. [6]—*A*, *n*, and *α*. *R* and *Q* represent the universal gas constant and activation energy, respectively. The amount of strain rate can be calculated by [31]:

(17) $$\dot{\varepsilon }={\left(\frac{2}{3}\left({\left(\frac{du}{dx}\right)}^{2}+{\left(\frac{dv}{dy}\right)}^{2}+{\left(\frac{dw}{dz}\right)}^{2}+\frac{1}{2}\left({\left(\frac{du}{dy}+\frac{dv}{dx}\right)}^{2}+{\left(\frac{du}{dz}+\frac{dw}{dx}\right)}^{2}+{\left(\frac{dw}{dy}+\frac{dv}{dz}\right)}^{2}\right)\right)\right)}^{\frac{1}{2}}$$

The properties of coolant were selected according to the water properties.

### 3.3. Water Flow Model

This simulation set the flowed water as a coolant by a separate domain with no chemical and mechanical interaction with base metal and joint line. Due to experimental results presented in the next section, here were voids and gaps inside and outside the joint line. However, due to the rotational velocity and forward motion of the FSW tool, the water flow cannot be steady. The water near the tool has a turbulent flow. This turbulent flow around the tool was modeled at a specific area around the FSW tool. The water (as coolant) imported to the welding setup has a steady-state flow. After the water reached the FSW tool, the water flow changed into turbulent flow, and after passing the tool, the water flow again changed into a steady flow.

**Figure 3:**
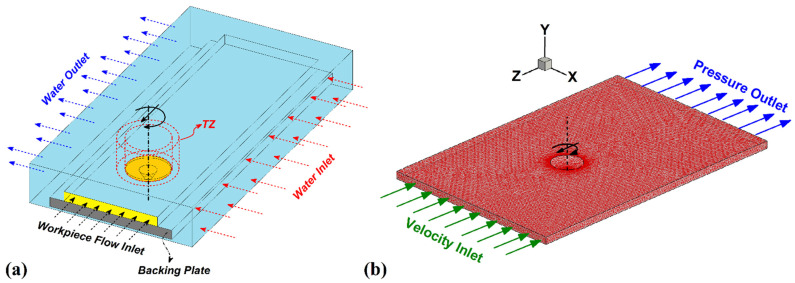
Isometric view of (**a**) water flow domain, (**b**) meshed domain for simulation of FSW and UFSW process.

For this reason, a surrounded area near the FSW tool was selected to study the turbulent flow of water, and at other areas, the water flow was considered steady. The simulation domain and turbulent zone (TZ) are depicted in Figure 3a. Accordingly, the turbulent flow in the TZ area can be defined as [32,33]:

(18) $$u(t)=\bar{u}+u^{\prime }(t)$$

(19) $$v(t)=\bar{v}+v^{\prime }(t)$$

(20) $$w(t)=\bar{w}+w^{\prime }(t)$$

The $\bar{u}$, $\bar{v}$, and $\bar{w}$ represent the mean velocity in *x*, *y*, and *z* directions. On the other hand, the $u^{\prime }(t)$, $v^{\prime }(t)$, and $w^{\prime }(t)$ are the turbulent fluctuation that are calculated by [34,35]:

(21) $$u^{\prime }(t)=u(t)+\int_{t}^{t+T}u(t)dt$$

(22) $$v^{\prime }(t)=v(t)+\int_{t}^{t+T}v(t)dt$$

(23) $$w^{\prime }(t)=w(t)+\int_{t}^{t+T}w(t)dt$$

### 3.4. Heat Transfer Model

This study used various heat transfer models due to various contact situations. As explained in the experimental procedure, the workpiece was fixed into the welding setup. In this situation, the backing plate was in with the bottom of the workpiece. For this reason, at the bottom surface, conductive heat transfer of weld metal with the backing plate is considered by:

(24) $$k{\left.\frac{\partial T}{\partial Z}\right|}_{Bottom}=h_{b}\left(T-T_{a}\right)$$

At the bottom face, the heat transfer coefficient has functioned on the local temperature through the following equation:

(25) $$h_{b}=h_{b0}{\left(T-T_{a}\right)}^{0.25}$$

On other hand, convective and radiation heat transfers were assumed as below at the top surface:

(26) $$-k{\left.\frac{\partial T}{\partial Z}\right|}_{Top}=Β\epsilon \left(T^{4}-T_{a}^{4}\right)+h_{t}\left(T-T_{a}\right)$$

For the complexity of the water phase (mixture of liquid and gas), the buoyancy-aided turbulent heat transfer model was used in the TZ area. For turbulent mixed convection in a uniform heated zone this model uses [36]:

(27) $$\frac{Nu}{Nu_{f}}={\left(\left|1\pm 8\times 10^{4}Bo^{*}{\left(\frac{Nu}{Nu_{f}}\right)}^{-2}\right|\right)}^{0.46}$$

In Equation (26), the positive and negative signs represented buoyancy-aided and buoyancy-opposite convection, respectively. The Nusselt numbers (*Nu* and *Nu_f_*) are mixed and forced convection. Bo* represents the buoyancy parameter to quantify the strength of buoyancy force. In this study, the Bo* is ~3 × 10^3^ because the value is usually used for buoyancy-aided mixed convection flow.

### 3.5. Boundary Conditions

In the FSW process, the heat is generated by frictional sliding contact (*F_h_*) between tool and workpiece and plastic deformation (*P_h_*) of AA6068-T6 aluminum alloy during angular velocity of the joining tool. Thus, the generated heat at the interfaces of AA6068 aluminum alloy and the FSW tool is defined by [37]:

(28) $$F_{h}=\left(\left(1-\beta \right)\chi \tau +\beta \mu_{f}F_{N}\right)\left(\omega r-v_{1}\sin\theta \right)\frac{A_{r}}{V}$$

(29) $$P_{h}=\psi \gamma \left(2\left({\left(\frac{\partial u}{\partial x}\right)}^{2}+{\left(\frac{\partial v}{\partial y}\right)}^{2}+{\left(\frac{\partial w}{\partial z}\right)}^{2}\right)+{\left(\frac{\partial u}{\partial y}+\frac{\partial v}{\partial x}\right)}^{2}+{\left(\frac{\partial u}{\partial z}+\frac{\partial w}{\partial x}\right)}^{2}+{\left(\frac{\partial w}{\partial y}+\frac{\partial v}{\partial z}\right)}^{2}\right)$$

In Equation (28), *u* represents velocity of materials in *x*, *y*, and *z* directions, and *ψ* is an arbitrary constant that indicates the mixing of materials in the stir zone. Due to the thermal conductivity between the aluminum alloy and the joining tool, the heat transferred to the welding tool should be considered. In this regard, the conduction heat transfer model is set on the interface of the FSW tool and aluminum alloy. During the simulation, the convection and radiation heat transfer model was set for the aluminum alloy’s top, sides, and bottom surfaces. The bottom surface of the base metal is connected to the welding fixture, and for this reason, the conduction heat transfer model is used for this area. The meshed domain is depicted in Figure 3b.

In a simulation procedure, the authors selected all process parameters, such as geometry, tool rotational speed, plunge depth, tilt angle, and traverse velocity according to the experimental design. During simulation, the tetrahedral/hybrid elements with a T-grid combination shape were selected as meshes for the welding tool and aluminum alloy workpiece in FSW and UFSW cases. The area near the welding tool required a much finer mesh to assess the plastic flow and heat transfer model. For this reason, to increase the simulation results’ reliability, a sizing function used on the welding tool and AA6068 aluminum alloy workpiece was used for generating the different volume mesh sizes. The sizing function in use contained three parameters: start size, maximum size, and growth rate. The start size was 0.05 mm for the fine mesh, the maximum size was 1.0 mm, and the growth rate was 1.1 mm. In the end, the total number of meshes for the FSW and UFSW cases was 3,124,467 volumes. 

ANSYS Fluent software was used to solve the governing equations. The simulation was tested to validate the experimental results. As a result, the simulation’s overall errors (when comparing experimental data) were less than 4%.

## 4. Results and Discussions

### 4.1. Thermal History and Material Flow

**Figure 4:**
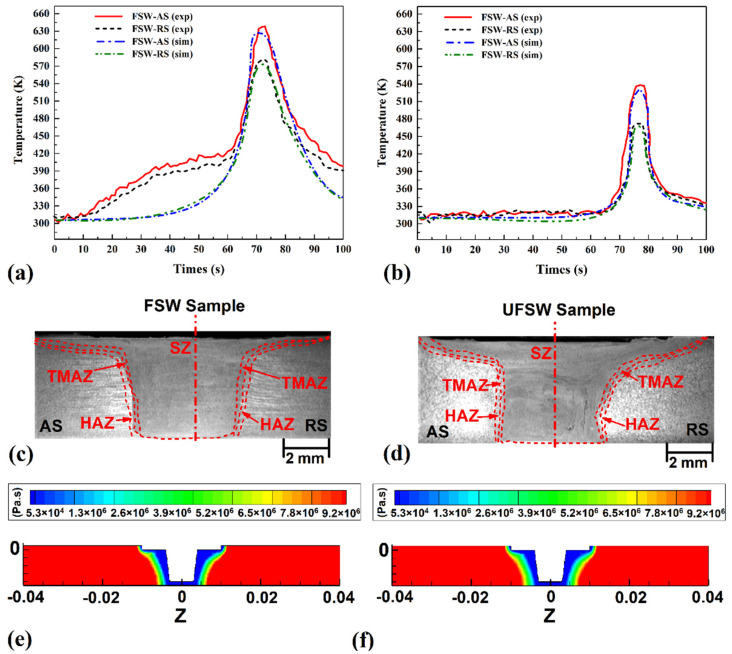
Experimental and simulation results from the thermal history of (**a**) FSW and (**b**) UFSW samples. Cross-section view of (**c**) FSW and (**d**) UFSW joints. Simulation results of materials viscosity changes in (**e**) UFSW and (**f**) FSW joint lines.

This section compares and analyzes the thermal history of FSW and UFSW joints with simulation results. The results obtained from the advancing side (AS) and retreating side (RS) are indicated in Figure 4a,b, respectively. 

As expected, the frictional heat generation was higher in AS than RS. The results show that generated heat in the FSW condition was more than UFSW and the cooling rate in UFSW was more than FSW. The maximum recorded frictional heat in AS was 658 K in conventional FSW and 549 K in UFSW condition. This thermal behavior changes the internal materials flow. The cross-section view of FSW and UFSW joints is indicated in Figure 4c,d. Due to the high cooling rate and low generated heat in the UFSW case, the stir zone (SZ) size is smaller than the FSW case. On the other hand, the SZ shape is not symmetrical. With the clockwise rotational of the tool and higher heat generation in the AS, the stir zone was formed bigger in the AS [38,39,40,41].

These materials’ flow behavior can be seen in both FSW and UFSW samples. The simulated result for the same condition was 650 K (FSW) and 540 K (UFSW) at AS, respectively. The simulation results of thermal history are presented in Figure 4a,b, respectively. By comparing, the experimental results indicate that the maximum heat generation in the RS was 571 K (FSW) and 485 K (UFSW), while the simulation results were 567 K (FSW) and 481 K (UFSW). This heat generation behavior affects the viscosity of the joint line during the welding procedure. Due to obtained results from simulation, higher heat generation in the FSW case comparing UFSW samples leads to more material softening. The simulation results of FSW and UFSW samples are depicted in Figure 4e,f, respectively. As seen, higher heat generation in FSW joint increased the softening area, the formed joint line in UFSW case is smaller than FSW sample.

**Figure 5:**
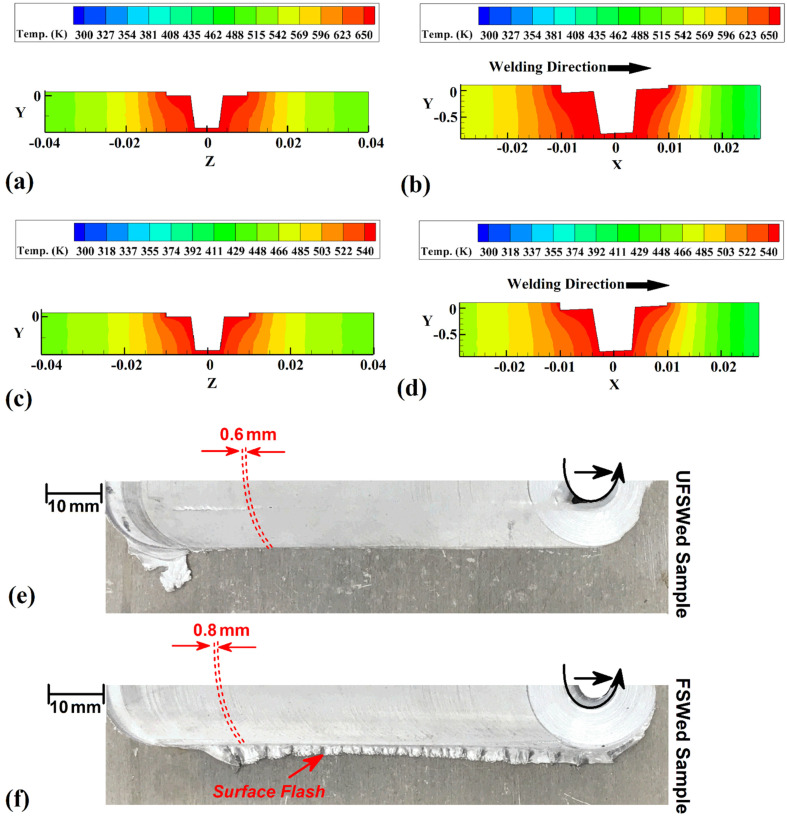
(**a**) cross-section and (**b**) longitudinal-section simulation results of internal heat flux of FSW sample. (**c**) Cross-section and (**d**) longitudinal-section simulation results of internal heat flux of UFSW sample. Surface materials flow of (**e**) UFSW and (**f**) FSW joint line.

The cross-section view (CSW) and longitudinal section view (LSW) of simulation results for FSW and UFSW joints are shown in Figure 5a–d, respectively. The simulation results indicate that the generated heat in FSW is higher than UFSW, and the hot area (which leads to the formation of stir zone (SZ)) in UFSW is smaller than FSW condition. The internal heat distribution in the FSW sample (Figure 5a,b) is more than the UFSW sample (Figure 5c,d). This phenomenon results from the higher cooling rate of water compared to air. Because the environment in the UFSW case is water and the heat transfer coefficient of water is higher than air, the internal heat flux of UFSW is lower than the FSW case. This behavior affects the surface flow of joints as well. The surface material flows of FSW and UFSW samples are depicted in Figure 5e,f, respectively. The surface flow in the UFSW case is smoother than in the FSW sample. The flow ring distance in the UFSW sample is closer than in the FSW case. The higher cooling rate and lower heat generation in the UFSW case improve the surface materials’ flow of the joint. Due to obtained results, the surface flash formed in the FSW sample was removed in the UFSW case, and the flow rings distance decreased from 0.8 (in the FSW case) to 0.6 mm (in the UFSW sample). This phenomenon seems to result from material velocity while stirring action and forward-moving of the FSW tool. The higher heat generation at FSW situation caused the plasticized materials from AS to RS to be transmitted rapidly, and during transverse moving of the tool, flow rings form with a higher flow ring distance. This behavior indicates that forming the joint line at the tool’s leading edge is more accessible than in the UFSW condition. In the SZ at the UFSW situation, the lower material velocity due to the lower heat generation makes material rotation harder.

### 4.2. Viscosity Changes and Material Velocity

**Figure 6:**
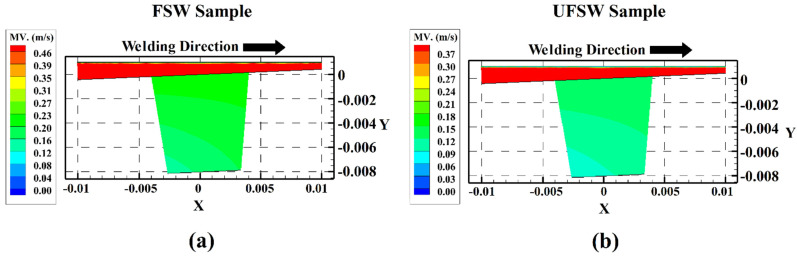
Side view of simulation results from materials velocity in (**a**) FSW and (**b**) UFSW samples.

Figure 6a,b indicate the simulation results of material velocity at SZ of the FSW sample and the UFSW sample, respectively. According to the results, the velocity of the materials is maximum around the exterior area of the FSW tool shoulder. Higher applied momentum caused this phenomenon. The origin point is placed at the tool axis, and the shoulder’s exterior edge produces higher momentum than the other part of the tool. This behavior is in agreement with experimental tests and is seen in both UFSW and FSW cases. By minimizing the tool radius near the tool axis, the momentum decays rapidly and decreases the velocity of material near the FSW tool axis. The simulation findings show that in the FSW situation, the mean material velocity is higher than in the UFSW case. According to the simulation results, the maximum velocity of material in SZ was 0.46 m/s at FSW and 0.37 m/s at UFSW conditions. The higher cooling rate and lower heat generation in underwater environments are the main reasons for decreased materials velocity.

### 4.3. Residual Strain

**Figure 7:**
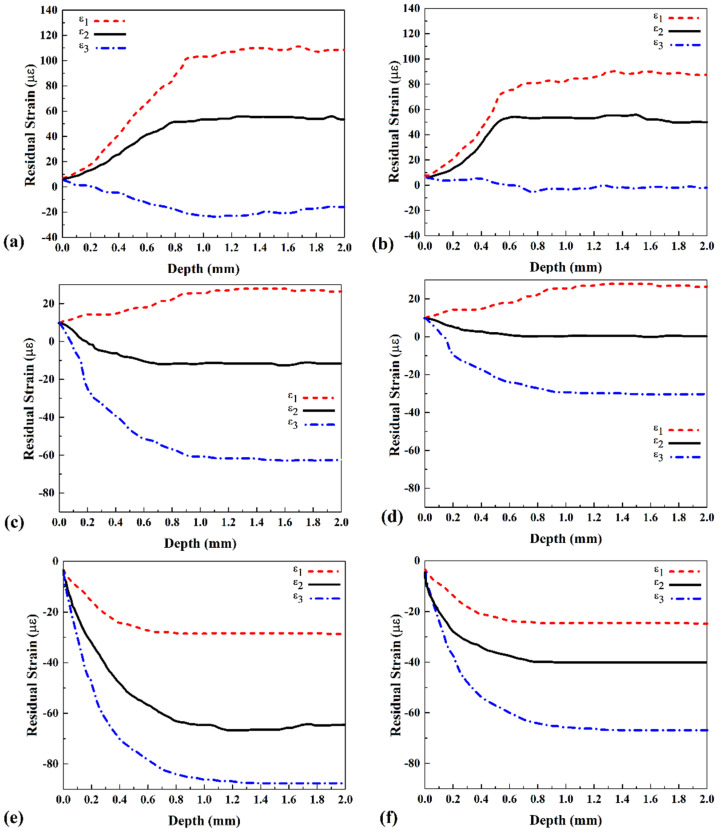
Measured residual strain in the FSW sample by (**a**) rosette (I), (**c**) rosette (II), and (**e**) rosette (III). Measured residual strain in the UFSW sample by (**b**) rosette (I), (**d**) rosette (II), and (**f**) rosette (III).

The residual stress in the welding area is tension, and in aluminum alloys, the residual stress is related to the base metal strength. Generally, with the increasing strength of weld metal, the residual stress increased after the welding process. The tensile strength of weld metal to the residual stress ratio (RSR) formed a specific number that can help understand residual stress on the weld line. This ratio could be different in a different part of the joint. For example, Lemmen et al. demonstrated that in FSW of AA7074-T6 aluminum alloy, the RSR in the stir zone (SZ) was 43%, and in the heat-affected zone (HAZ) was 62%. Due to softening of the HAZ area after FSW, the RSR in the HAZ area increased, and this result shows that the RSR in the HAZ area is much more defective than the SZ area. The statistical results of released strain in different strain gauges are depicted in Figure 6. The results consist of data provided by rosette (I), (II), and (III) in the FSW case, presented in Figure 7a–e, respectively. 

Furthermore, the results consist of data provided by rosette (I), (II), and (III) in the UFSW case, presented in Figure 7b–f, respectively. All figures consist of three lines. These lines show residual strain in direction ε1 (x-direction), direction ε2 (y-direction), and direction ε3 (z-direction). The figures’ bars show the depth of the drilling hole at each rosette and the amount of residual strain. Due to obtained results, the residual strain in rosette (I) is higher than in other places. According to the rosette’s placements, the residual strain in the joint line is higher than in other areas. In both FSW and UFSW cases, this behavior can be observed. As the results show, the released strain in the FSW sample (Figure 7a) increased from the surface until 0.8 mm depth of the hole, and from a depth more than 0.8 mm, the released strain became stable. Meanwhile, released strain in the UFSW sample (Figure 7b) increased from surface until 0.6 mm depth of the hole and after that became stable. This trend is expected from the CHD process because the maximum RSR measurement is at 2 mm hole depth. The residual strain in the x-direction (ε1) is higher than in other directions, and the residual strain in the z-direction (ε3) is lowest. This trend followed at all rosettes’ places and welding cases. As the results show, the released strain in the UFSW case is lower than in the FSW. Due to the faster cooling rate and lower heat generation in submerged cases, the RSR in the UFSW case is lower than in the FSW sample. The maximum strain in FSW and UFSW joint lines was ~110 µε and ~92 µε, respectively. With distance increasing from rosette (I), the released strain decreased in both cases.

The ε1 at 15 mm and 30 mm from the joint line in the FSW sample were 30 µε and −28 µε, while the ε1 at 15 mm and 30 mm from the joint line in the UFSW sample were 25 µε and −22 µε, respectively. The minus sign of residual strain shows that the strain in the joint line is stretching, and with increasing from the joint line, it became compression. The results revealed that the residual strain of ε3 at 15 mm and 30 mm from the joint line in the FSW sample was −26 µε and −95 µε, while the ε3 at 15 mm and 30 mm from the joint line in the UFSW sample were −10 µε and −65 µε, respectively. By comparing the results, it will be apparent that the different amount of residual strain between ε1 and ε3 in the UFSW case is much lower than the FSW sample.

### 4.4. Residual Stress

**Figure 8:**
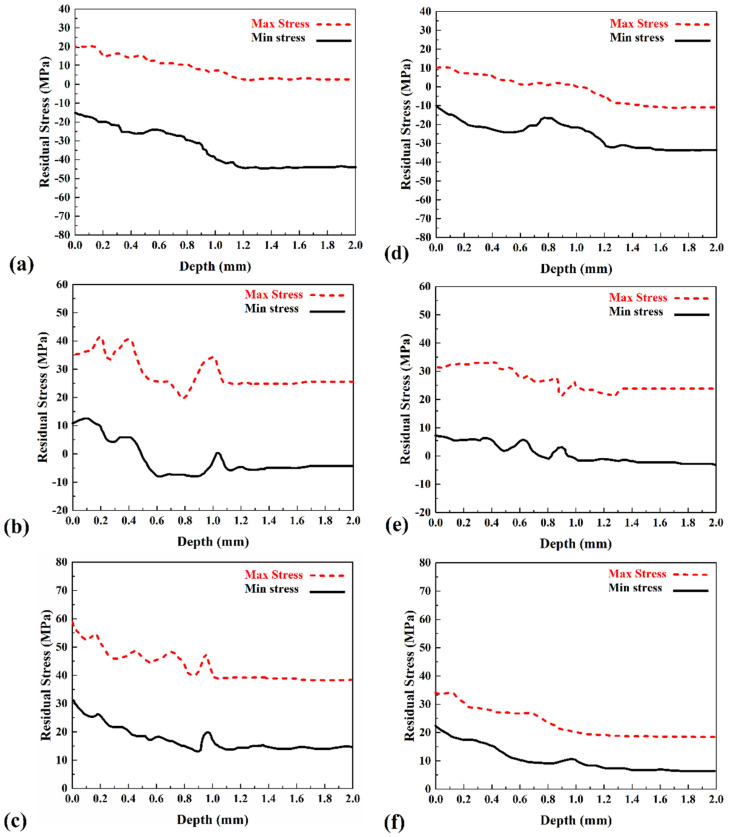
Calculated residual stress in the FSW sample by (**a**) rosette (I), (**c**) rosette (II), and (**e**) rosette (III). Calculated residual stress in the UFSW sample by (**b**) rosette (I), (**d**) rosette (II), and (**f**) rosette (III).

According to the ASTM-E837-13a standard number, the maximum stress determined by the CHD method is half of the base metal. By solving Equation (10), the maximum and minimum stress for all rosettes were calculated, and the results are presented in Figure 8.

Due to the obtained result, the residual stress was not more than half of the base metal strength. The results of residual stress are provided by data from rosettes (I), (II), and, (III) in the FSW case, presented in Figure 8a–e, respectively. Furthermore, the residual stress results provided from rosettes (I), (II), and (III) in the UFSW case are presented in Figure 8b–f, respectively. All figures consist of two lines that show the maximum (dash line) and minimum (solid line) residual stress along drilled holes.

The obtained results revealed that the maximum and minimum stresses in the joint lines are tension stress, and with increasing distance from the joint line, the stress increased smoothly and decreased after that. Comparing the FSW and UFSW cases revealed that the stress in the FSW case is more than the UFSW joint. The average maximum and minimum residual stress in FSW joint line were 20 MPa and −50 MPa, and the average maximum and minimum residual stress in the UFSW joint line were 8 MPa and −20 MPa. The average maximum and minimum residual stress at 30 mm from the FSW joint line were 49 MPa and 15 MPa, while the average maximum and minimum residual stress at 30 mm far from the UFSW joint line were 21 MPa and 13 MPa. With a comparison between residual stress, it can be found that the average maximum residual stress in the UFSW joint line is 12 MPa lower than the FSW sample. The average minimum residual stress in the FSW case is 30 MPa more than the UFSW case. On the other hand, the difference of residual stress (average maximum) in the FSW joint line with a 30 mm more extended area is near 29 MPa, and this number in the UFSW case is 13 MPa. These numbers indicated that the residual stress in the UFSW sample is much lower than the FSW sample.

## 5. Conclusions

This article considers friction stir welding and underwater friction stir welding of AA6068-T6 aluminum alloy. The processes simulated by the CFD method and thermal history, materials flow, residual strain, and residual stress are evaluated in both cases. The conclusion of this research can be presented as:

1. The heat generation in the UFSW sample was lower than FSW, which caused the joint line formed in the UFSW sample to be smaller than the FSW case. The cooling rate of the AA6068-T6 aluminum joint line in the UFSW case was higher than the FSW case.
2. The simulation results revealed that materials velocity in the SZ of the FSW sample (0.46 m/s) is 24% higher than the UFSW (0.37 m/s) sample. This phenomenon is related to higher heat generation in the FSW case, and as a result, the surface flow ring distance in the FSW sample (0.8 mm) is 25% higher than the UFSW (0.6 mm) sample.
3. With increasing distance from the joint line, both residual stress and residual strain increase. This phenomenon is seen in both FSW and UFSW samples. Due to obtained results, the residual stress in the ε1 direction is more than ε2 and ε3 directions.
4. The obtained result revealed that the maximum residual strain (ε1) in the FSW joint line (30 µε) is 20% more than the UFSW joint line (25 µε). On the other hand, the maximum residual stress (ε1) in the UFSW joint line (8 MPa) is 60% lower than the FSW joint line (20 MPa). The higher cooling rate and controlling of heat generation in an underwater environment improve the residual strain and stress of the UFSW joint.

## Figures and Tables

**Table 1:** Mechanical properties of base metal.

Parameter	Density	Thermal Conductivity	Ultimate Tensile Strength	Shear Stress	Elongation	Vickers Hardness
Unit	kg/m^3^	W/m.K	MPa	MPa	%	HV
Amount	2700	180	300	460	10	91

## Data Availability

Data sharing is not applicable to this article.
